# 特发性肺纤维化相关的肺癌发病机制的研究进展

**DOI:** 10.3779/j.issn.1009-3419.2022.101.51

**Published:** 2022-11-20

**Authors:** 蔷薇 卢, 闪 韩, 晓秋 刘

**Affiliations:** 130000 长春，吉林大学第二医院呼吸与危重症医学科 Department of Respiratory and Critical Care Medicine, Second Hospital of Jilin University, Changchun 130000, China

**Keywords:** 特发性肺纤维化, 肺肿瘤, 细胞, 分子, 发病机制, Idiopathic pulmonary fibrosis, Lung neoplasms, Cell, Molecular, Pathogenesis

## Abstract

特发性肺纤维化（idiopathic pulmonary fibrosis, IPF）是一种原因不明的最常见的间质性肺疾病（interstitial lung disease, ILD），以最终导致肺间质纤维化为特征，确诊后的中位生存期为2年-4年。近年来，IPF相关的肺癌（lung cancer associated with IPF, IPF-LC）的发病率越来越高，且预后较单纯的IPF差。研究发现，IPF可能与肺癌的发生发展密切相关。尽管IPF-LC的发生机制尚不清楚，但目前的研究表示这两种疾病在分子及细胞水平的致病过程存在相似之处。目前肺纤维化相关的肺癌的细胞分子机制的研究已经成为研究者的关注焦点。本文通过回顾相关文献，以IPF-LC的细胞和分子机制及治疗最新现状为重点进行综述，希望能够提高临床医生对IPF-LC的认识。

## Abstract (en)

Idiopathic pulmonary fibrosis (IPF) is the most common interstitial lung disease (ILD) of unknown causes, which is characterized by pulmonary fibrosis. The median survival period after diagnosis is about 2-4 years. In recent years, the incidence rate of lung cancer associated with IPF (IPF-LC) is increasing, and the prognosis is worse than that of IPF alone. Pulmonary fibrosis may be closely associated with the occurrence and development of lung cancer. Although the pathogenesis of IPF-LC is still unclear, the current research shows that there are similarities between the pathogenesis of these two diseases at molecular and cellular levels. At present, the research on the cellular and molecular mechanism of lung cancer related to pulmonary fibrosis has become the focus of researchers' attention. This article reviews the related literature, focusing on the latest status of the cellular and molecular mechanisms and treatment of IPF-LC, hoping to help clinicians understand IPF-LC.

特发性肺纤维化（idiopathic pulmonary fibrosis, IPF）是一种病因不明的慢性、进行性、纤维化性间质性肺炎。早在1965年就有报道提出肺纤维化与肺癌（lung cancer, LC）相关^[1]^，鉴于这两种疾病存在多种共同的遗传、细胞和分子过程，因此这可能使患者容易合并发生IPF和LC^[2]^。IPF是间质性肺疾病（interstitial lung disease, ILD）最常见的类型，而LC是ILD患者，尤其是IPF患者的一种重要并发症^[3]^。在临床上，患者同时患有ILD和LC是很常见的。许多研究也提示IPF与LC在表观遗传、遗传变化以及致病序列方面存在相似性，也具有增殖失控、特异性信号通路异常和microRNAs异常表达等共同特征^[4]^。因此，LC和IPF之间存在密切关联。

## 1 IPF和LC的相关性

IPF是临床上最常见的一种特发性间质性肺炎。目前临床上出现了越来越多的IPF-LC的病例。现在的观点认为IPF本身是LC发生的独立危险因素，关于IPF-LC也没有明确的诊断和预后生物标志物。IPF发病机制的潜在分子变化具有促进肿瘤发生的作用。许多IPF患者存在危险因素，例如吸烟史或伴发肺气肿，并且这两者都可能使IPF患者更易患LC，特别是非小细胞肺癌（non-small cell lung cancer, NSCLC），而IPF本身也会增加7%-20%的LC发病风险^[2]^。

### 1.1 流行病学

IPF在ILD中占有特殊的地位，其病程不可逆转，目前还没有特效药物逆转其病程。IPF从诊断时起，中位生存期仅为2年-4年，预后甚至比许多癌症差。一项针对IPF全球发病率和患病率的研究^[5]^表明IPF调整后发病率和患病率分别在0.09-1.30/10, 000人和0.33-4.51/10, 000人的范围内。而LC与IPF患者生存期恶化有关。流行病学证据^[6]^表明，IPF患者的LC发病风险最高，并在老年男性吸烟者中更为常见。Ballester等^[2]^报道了IPF中LC的患病率为2.7%-48%，明显高于一般人群，同时也表示LC患病率在不同地区存在差异，亚洲的患病率为15.3%，欧洲则为11.6%。

随着IPF患者随访时间的延长，LC的累计发病率显著上升。研究^[7]^表明，随着IPF病程的进展，LC的发病率在1年时为1.1%，3年时为8.7%，5年时为15.9%，10年时为31.1%。

### 1.2 IPF导致LC的危险因素

IPF与LC之间的关联之前已有报道，但与IPF-LC发展相关的发病率和临床因素尚不清楚。多年来，研究者^[7-10]^通过对大量初始诊断为单纯IPF患者经过随访及系列研究得出：吸烟、男性、老龄化、FVC迅速恶化、合并肺气肿是目前公认IPF发生LC的危险因素。了解这些因素有助于指导临床医生对LC的早期发现以及对疾病更加规范化管理。

### 1.3 IPF-LC患者的临床特征

LC是IPF常见的合并症之一，影响患者的生活质量和预后。LC的类型以NSCLC为主，其中以腺癌多见。而IPF-LC的病理学分型以NSCLC多见，大多数研究^[7, 11-13]^表明主要类型为鳞状细胞癌，但也有研究^[4]^表明腺癌更多见，且多位于IPF受累区域。有研究^[4]^还发现IPF-LC患者的胸痛、咯血和消瘦症状比单纯IPF患者更常见；癌胚抗原（carcinoembryonicantigen, CEA）和癌抗原125（cancer antigen 125, CA125）水平在IPF-LC患者中更高，胸部高分辨率计算机断层扫描（high resolution computed tomography, HRCT）上位于外周和下叶的非典型淋巴结或肿块也更多见于IPF-LC患者。因此，当IPF患者在原有临床表现上出现胸痛、咯血、消瘦加重，血液检查指标，例如CEA和CA125较前明显升高，影像学检查在原有基础上出现肿块以及非典型淋巴结，应高度警惕合并LC的可能性。IPF患者的LC发病在肺下叶发生率更高（*P* < 0.001），而非IPF-ILD患者在LC的发生定位方面与一般人群没有差异^[14]^。

近期有学者对IPF患者影像学中普通型间质性肺炎（usual interstitial pneumonia, UIP）和非UIP型与LC的关系进行了研究，发现在IPF患者中，LC通常发生在肺纤维化区域，正常的肺组织已被纤维化和蜂窝状结构所取代。IPF在影像学上通常表现为UIP。有研究^[15]^表明在UIP/IPF-LC患者中，癌症在具有蜂窝状或移行性成纤维细胞病灶的区域的肺外周和晚期纤维化环境中发展，致癌机制与IPF中的纤维化和炎症过程密切相关，而与非UIP-ILD（特别是吸烟相关型ILD）无关。

## 2 IPF-LC的发病机制

IPF患者的LC患病率和发病率增加提示IPF本身可能促进LC的发展^[2]^。除了基因和表观遗传学改变，IPF发展过程中的一些分子和细胞过程促使IPF-LC的发生。

肺纤维化是成纤维细胞（主要是肌成纤维细胞）积聚和持续存在引起的。与IPF相似，肿瘤被描述为“无法愈合的损伤”，且大量文献支持损伤修复和癌症的重叠发生机制。在这些机制中，肿瘤基质和肿瘤相关成纤维细胞起关键作用^[16]^。一些细胞和分子过程将肺纤维化与LC联系起来，例如肌成纤维细胞-间充质转化（epithelial to mesenchymal transformation, EMT）、肌成纤维细胞活化以及不受控制的增殖、内质网应激、生长因子表达的改变、氧化应激和大的遗传和表观遗传变异等。

### 2.1 成纤维细胞增殖、分化和EMT

IPF的病理特点是纤维化肺组织中大量的成纤维细胞和肌成纤维细胞持续活化，活化后会分泌大量的细胞外间质（extracellular matrix, ECM）成分。EMT是指上皮细胞通过特定程序转化为具有间质表型细胞的生物学过程，并在胚胎发育、慢性炎症、组织重建、癌症转移和多种纤维化疾病中发挥了重要作用。一种理论认为组织损伤在转化生长因子-β（transforming growth factor-beta, TGF-β）的存在下诱导EMT^[17, 18]^，TGF-β1在正常上皮细胞和癌细胞中均诱导EMT。EMT是伤口愈合的一种形式，在这种形式中，如果引发伤口愈合的原发炎性刺激不能消失或减弱，II型肺泡上皮细胞可以作为肌成纤维细胞的来源，最终导致器官破坏。研究^[19]^发现正常的外周肺上皮细胞可以在72 h内发生动态EMT以响应TGF-β1信号传导，这一发现提示经历EMT的肺泡上皮细胞促进成纤维细胞灶的形成，成纤维细胞是肺部纤维化破坏的前沿。与IPF相似，癌症相关成纤维细胞（cancer-associated fibroblasts, CAF）也是LC中的重要参与者，因为CAF也表现出间充质样特征，并具有异质性表型；在癌变、癌症侵袭和转移的早期阶段，驻留上皮细胞（通常为细支气管上皮细胞）也可能经历可逆的EMT。而从外周循环招募的纤维细胞也被认为是CAF的潜在来源^[16]^。CAF通过与癌细胞的相互作用促进肿瘤上皮间质转化，据报道CAF也通过诱导EMT表型促进肺腺癌侵袭和转移^[20]^。EMT逐渐成为与抗癌药物联合使用的抗癌治疗的焦点^[21]^。

### 2.2 细胞凋亡、自噬以及内质网（endoplasmic reticulum, ER）应激

细胞凋亡是一种由基因控制的自主有序的死亡，可以维持体内平衡，而IPF的肺成纤维细胞凋亡减少。自噬负责通过降解细胞器和大分子来回收各种成分，为重建细胞结构提供原材料，维持成纤维细胞的正常寿命，而IPF患者肺组织中的自噬活性降低^[22]^。自噬和细胞凋亡也在LC进展过程中起重要作用。一方面，自噬被不同的癌蛋白抑制，如AKT、PI3K、Bcl-1和突变*p53*，这可能会阻止应激的肿瘤细胞中蛋白质的过度降解。另一方面，自噬的持续激活会导致自噬性程序性细胞死亡或凋亡，研究^[23]^表明，ER应激是细胞凋亡和自噬的关键调节因子。ER应激在IPF中起着显著作用，可以引起肺泡上皮细胞功能障碍和肺纤维化，并导致未折叠蛋白反应（unfolded protein response, UPR）^[24, 25]^。而UPR可以改善蛋白质折叠，维持细胞稳态，并防止细胞因错误折叠蛋白质的积累而死亡，这些蛋白质会聚集并干扰基本细胞功能；UPR还可通过减少蛋白质翻译、增加代谢和氧化还原蛋白表达、增强ER伴侣产生以及促进蛋白质降解的机制发挥作用。当UPR机制失效或ER应激过于严重时，可能会通过细胞凋亡导致生长停滞和细胞死亡^[24]^。针对于LC，研究^[23]^表明一些植物化合物具有抗癌作用，且大多数通过诱导ER应激发挥作用。然而有证据^[26]^表明，ER应激可能减弱衰老并促进肿瘤发生，增加的ER应激在一定程度上通过上调磷酸化蛋白激酶B（phosphorylated protein kinase B, p-AKT）和减少磷酸化细胞外信号调节激酶（phosphor-extracellular signal-regulated kinase, p-ERK）来减弱衰老，p-AKT、ER应激和UPR之间存在正前馈循环，ER应激的瞬时增加导致衰老减少，最终促进肿瘤发生。

### 2.3 细胞间通讯异常

研究^[27]^已经证实细胞间通讯途径能够促进肺部发挥相应功能来应对损伤和疾病。这些途径涉及连接蛋白（connexins, CXS）和缝隙连接细胞间通讯（gap junctional intercellular communication, GJIC），而引发LC的外源性因素均与CXS在肺中表达的位置异常或缺失有关，例如在人NSCLC样本中，连接蛋白43（Cx43）的表达就很低，因此细胞间通讯异常可能会促进肿瘤的发生。GJIC在许多生物过程中起着关键作用，包括伤口修复和促纤维化。研究者使用啮齿类动物来源的腹膜肥大细胞系（retinal muller cell line, RMC-1）和人皮肤来源的成纤维细胞建立体外模型，研究CX43的表达对不同细胞GJIC、微管β-微管蛋白和微丝α-平滑肌肌动蛋白（α-smooth muscle actin, α-SMA）表达的影响，得出在不表达CX43的情况下，成纤维细胞表达其他参与GJIC的CXS；RMC-1细胞与成纤维细胞间的不同细胞间连接可将成纤维细胞转化为肌成纤维细胞，在胞浆应激纤维内表达α-SMA。CX43在RMC-1细胞中被敲除后，β-微管蛋白的表达增加；而在成纤维细胞中被敲除后，β-微管蛋白的表达减少。下调CX43在成纤维细胞中的表达可抑制α-SMA的表达。Cx43的参与对于肥大细胞和成纤维细胞之间的不同细胞间的GJIC至关重要，这可能为控制纤维化提供新的研究方向^[28]^，细胞间通讯途径及其异常反应也可能为肺纤维化及LC的治疗提供新的靶点。

### 2.4 细胞衰老

Hashimoto等^[29]^的研究表明细胞衰老会促进肺部老化，且可能成为改善肺功能的研究目标。衰老是IPF最重要的危险因素之一，流行病学资料证实IPF是一种与衰老相关的疾病。IPF中衰老的成纤维细胞表现出异常激活、端粒缩短、代谢重编程、线粒体功能障碍、凋亡抵抗、自噬能力下降和衰老相关分泌表型（senescence-associated secretory phenotype, SASP）。细胞衰老是一种在进化上保守的稳定复制停滞状态，损伤积累会激活细胞周期蛋白依赖性激酶抑制剂p16Ink4a和/或p53-p21Cip1/Waf1，二者拮抗细胞周期蛋白依赖性激酶并阻断细胞周期进程。而且衰老细胞通过分泌SASP，即一种广泛的细胞因子、趋化因子、基质重塑蛋白酶和生长因子，能够旁分泌促进增殖和组织退化。衰老细胞通过SASP对相邻细胞产生有效影响，最终促进肺功能恶化^[30]^。有学者^[31]^报道通过在体内靶向衰老成纤维细胞来增加NSCLC放疗的敏感性，并能够减轻辐射诱导的肺纤维化。但在癌症中关于针对细胞衰老的治疗是有争议的。一方面，细胞衰老可能限制细胞的复制能力，并阻止恶性肿瘤在不同阶段的细胞增殖，同时为肿瘤扩展提供防御屏障；另一方面，有人提出衰老成纤维细胞可能通过分泌SASP来促进肿瘤进展^[32]^。细胞衰老及SASP在与衰老相关的IPF和LC的发病机制中起关键作用，许多SASP因子，如由癌基因诱导的白介素-6（interleukin-6, IL-6）和IL-8也会促进恶性转化^[33]^。因此，细胞衰老为IPF-LC的发生机制及治疗提供了新方向。

### 2.5 炎症反应与组织侵袭

炎症细胞和细胞因子参与IPF-LC的发生发展。研究^[34]^表明M2巨噬细胞是IPF-LC发展中的关键效应物。IPF中存在铁粒相关巨噬细胞，也是一种M2表型的巨噬细胞。目前，M2巨噬细胞已被确定为肿瘤发展的触发细胞。肿瘤相关巨噬细胞也表达M2表型，并通过促进血管生成、激活间充质细胞、重塑基质和抑制效应T细胞反应促进肿瘤生长^[2]^。未来的研究可以着重于M2巨噬细胞触发肿瘤发生的机制。TGF-β1是IPF发病和致癌的重要介质。在肿瘤微环境中，来自癌源性上皮细胞的TGF-β1在癌组织的侵袭性前沿诱导肌成纤维细胞募集，并保护肌成纤维细胞免于凋亡。这些肌成纤维细胞包围肿瘤组织并产生TGF-β1，借助炎症介质和金属蛋白酶，肌成纤维细胞会破坏周围组织的基底膜并促进肺部肿瘤侵袭^[35]^；在IPF中，肌成纤维细胞通过自分泌TGF-β1促进细胞增殖，最终导致增殖失控，促进肿瘤的发生发展^[36]^。

### 2.6 致纤维化分子

大量研究证明，致纤维化分子例如：TGF-β1、酪氨酸激酶受体配体血小板衍生生长因子、血管内皮生长因子和成纤维细胞生长因子、溶血磷脂酸、趋化因子配体2[chemokine (c-c motif) ligand 2, CCL2]、IL-13等^[2]^共同参与IPF和LC的发病过程。成纤维细胞在纤维化区的扩张需要成纤维细胞生长因子信号^[37]^，而成纤维细胞生长因子还可以影响LC细胞的增殖、治疗敏感性和凋亡^[38]^。致纤维化分子同时参与纤维化的形成以及肿瘤的发生，因此对于参与IPF-LC细胞因子的深入研究有助于为治疗提供新靶点。

### 2.7 细胞信号转导机制/信号通路的异常激活

IPF与LC之间存在关联。与非IPF相关的LC相比，IPF患者的LC优先在紧邻纤维化区域的外周发展。研究^[39]^表明IPF和LC具有致病相似性，包括遗传和表观遗传改变，而这些遗传和表观遗传改变导致了常见转导通路的异常激活，包括Wnt/β-Catenin信号通路、PI3K/Akt/mTOR通路以及细胞程序性死亡蛋白1（programmed death protein 1, PD-1）及其配体程序性死亡配体1（programmed death ligand 1, PD-L1）通路激活等。

Wnt通路与一些纤维化疾病的发病机制有关，而有证据表明β-Catenin信号传导在诱导EMT中发挥作用，这是在胚胎发育、肿瘤进展和损伤后纤维化组织修复的关键阶段的重要过程^[40]^。根据细胞内β-Catenin的参与程度，Wnt信号通路主要分为两类：经典的β-Catenin非依赖性通路和非经典的β-Catenin非依赖性通路。研究^[41]^表明，Wnt配体的上调对衰老肺组织具有额外作用，可以介导肌成纤维细胞相关标志物的表达。在鼠模型中，Wnt信号通路的激活与肿瘤起始潜能增加有关，并且越来越多的证据表明Wnt通路在NSCLC的发展中很重要。Wnt信号传导很复杂，可能与其他几种途径相互作用，并产生可能促进肿瘤侵袭性以及抑制肿瘤侵袭性的因子，除了Wnt-7a信号传导之外，Wnt通路的总体影响似乎是促进肿瘤侵袭性和对化疗和放疗的抵抗力^[42]^。

PI3K/Akt/mTOR通路是肺纤维化发生机制的经典信号通路，PI3K/AKT/mTOR信号通路的异常激活可以增加成纤维细胞的增殖，并减少自噬和细胞凋亡^[43]^，最终引起纤维化的发生。PI3K/AKT/mTOR通路的失调也与NSCLC有关，特别是与高级别肿瘤和疾病晚期相关^[44]^。PI3K/Akt/mTOR通路激活后能够增加许多致癌特征，如获得性生长信号的自主性、抑制凋亡、持续血管生成、增加组织侵袭和转移以及对抗生长信号不敏感^[45]^。

PD-1通路被癌细胞用来逃避免疫系统的监视，使用单克隆抗体阻断PD-1/PD-L1可为各种癌症患者带来益处。而阻断免疫检查点的临床研究的发展极大地改变了NSCLC患者的治疗模式和预后，PD-1和PD-L1抗体的免疫检查点阻断在晚期NSCLC患者中产生具有临床意义的持久反应^[46]^。一项研究^[47]^发现，与健康对照组相比，肺纤维化患者肺组织中的CD4^+^和CD8^+^细胞以及外周血中的PD-1^+^淋巴细胞增加。另一报道^[48]^表明，PD-1在CD4^+^ T细胞中上调并促进博莱霉素诱导的肺纤维化。研究者对侵袭性和非侵袭性成纤维细胞进行了RNA测序分析，发现免疫检查点配体CD274（也称为PD-L1）在侵袭性肺成纤维细胞中上调，并证明在IPF的成纤维细胞中激活PD-L1可促进体外侵袭和体内肺纤维化^[49]^。因此，阻断PD-1/PD-L1通路激活是抑制肺纤维化的新方向。

Notch信号是一种高度保守的细胞间信号通路，其通过与相邻细胞直接接触来传输信号，因此适合短距离的细胞通讯。研究^[50]^表示Notch信号通路在呼吸系统的发育、体内平衡和再生过程中起着关键作用。Notch通路与IPF有关，在IPF患者的高度纤维化肺泡区域中Notch1通路成分的表达显著增强^[51]^，表达Notch1的细胞还表达肌成纤维细胞的标志物SMA^[50]^。研究^[52]^称Notch信号传导参与肿瘤发生，小鼠肺腺癌模型中的Notch1缺失明显降低了肺腺癌的发生，并从分子水平解释了Notch1表达与NSCLC患者预后不良之间的关联。另外，小鼠肺中Notch信号过度表达会抑制神经内分泌细胞分化，Notch信号可以通过抑制神经内分泌标志物在人类SCLC中充当肿瘤抑制因子^[50]^。

## 3 治疗进展

目前，关于IPF-LC的诊断和预后生物标志物尚不清楚。IPF-LC的治疗应该兼顾IPF和LC的治疗，结合IPF分期、LC分期及病理分型，并应该采取个体化原则治疗方案。目前仍缺乏IPF-LC治疗的前瞻性随机研究，有关IPF-LC患者应怎样选择合适的治疗手段仍在探索中。

### 3.1 手术治疗

手术是治疗早期LC的主要方式，但IPF-LC患者肺切除术后发生IPF加重和死亡的风险较高。IPF患LC的风险更高，因为这两种疾病具有共同的风险因素。然而，由于IPF的罕见发病率、预后不良和治疗期间的急性加重（acute exacerbation, AE），尚无针对IPF-LC的标准治疗方式。有研究根据LC分期和GAP[性别（G）、年龄（A）和两个肺生理变量（P）]分期确定LC治疗的效果和IPF患者的预后，最终得出在GAP I期，手术能够使LC患者有更好的生存率；在GAP II期/III期中，没有任何治疗方式能显著改善临床结果。GAP I期应考虑积极治疗，晚期GAP期患者在决定是否手术时应考虑体能状态和LC分期^[53]^。总之，手术治疗对于IPF-LC患者的要求较高，且预后不明。

### 3.2 药物治疗

#### 3.2.1 化学药物治疗

卡铂联合紫杉醇是ILD的NSCLC患者的一线治疗（61%）方案^[54]^。IPF-LC患者由于化疗而发生AE-IPF的风险为10%-30%^[53]^。鉴于IPF自然病程中每年的AE发病风险为5%-15%，并且未合并IPF的患者的药物诱导性肺炎的风险通常低于5%，用化疗药物治疗IPF-LC的发生率更高。有研究^[55]^表明，吡非尼酮联合以卡铂为基础的方案或免疫检查点抑制剂可能是IPF和NSCLC患者的比较安全的一线化疗方案，在LC治疗过程中占绝大部分。

#### 3.2.2 靶向治疗

EGFR酪氨酸激酶抑制剂吉非替尼在治疗LC后会发生药物性间质性肺炎，因其严重性和死亡率，已经成为一个严重的社会问题。发生药物性间质性肺炎的患者中ILD相关的死亡占吉非替尼治疗患者的31.6%，占化疗患者的27.9%^[56]^。

#### 3.2.3 免疫治疗

用于LC治疗的纳武利尤单抗作用于PD-1，已用于IPF-LC患者。Duchemann等^[57]^报道3例轻中度肺间质纤维化合并LC患者应用纳武利尤单抗治疗后，随访期间无一例发生肺部毒性损伤或肺间质纤维化恶化的情况，其死亡原因与LC进展有关。最近获批的一种治疗药物罗伐匹单抗，目前用于小细胞肺癌（small cell lung cancer, SCLC），但在IPF和IPF-LC患者中也可能发挥作用，它能够在博莱霉素诱导肺纤维化大鼠模型中干扰和抑制Notch信号通路，从而改善肺纤维化^[58]^。在未来的研究及临床试验中，PD-1抑制剂相关药物可能为IPF-LC带来新方法。

### 3.3 抗纤维化治疗在IPF-LC中的作用

吡非尼酮（Pirfenidone）和尼达尼布（Nintedanib）是目前唯一获批的抗纤维化药物。由于ILD和LC的发病机制存在重叠途径，理论上抗纤维化治疗可能具有抗肿瘤作用。已有报道^[59]^表示抗纤维化药物能够减少IPF-LC患者手术治疗后，患者AE-IPF发病率方面的益处。Nintedanib是酪氨酸激酶抑制剂，具有抑制蛋白激酶的潜力，这些蛋白激酶涉及导致癌症和纤维化发生和发展的几种分子途径。因为Nintedanib可以抑制血管生成，最初是作为一种抗癌药物存在，研究发现其又具有抗纤维化作用，又被批准用于治疗IPF^[60]^。Nintedanib下调ECM蛋白、纤连蛋白和胶原蛋白1a1的蛋白和mRNA表达，同时抑制TGF-β1诱导的肌成纤维细胞分化，并诱导Beclin-1依赖、ATG7非依赖的自噬，抑制TGF-β信号传导的早期事件，特别是II型TGF-β受体的酪氨酸磷酸化、SMAD3的激活和p38丝裂原活化蛋白激酶^[61]^。目前Nintedanib联合多西紫杉醇已被批准为二线抗癌方案，用于一线化疗后局部晚期、转移或局部复发的腺癌成年患者^[62]^。有研究^[63]^也表示接受抗纤维化治疗的IPF患者的LC发生率和患病率明显低于未接受抗纤维化治疗的患者，接受抗纤维化治疗的IPF患者的LC相关死亡率显著降低，抗纤维化治疗可能与IPF患者发生LC的风险降低有关。Pirfenidone和Nintedanib在IPF和LC的治疗方面都取得了较好的效果，但未来仍需要进一步研究该两种药物在IPF-LC的效果。Plumbagin（PL）是一种具有多种药理作用的草药提取物，被用于治疗各种类型的癌症，研究^[64]^表明PL可以作为一种抗纤维化剂，通过下调TGF-β1/结缔组织生长因子（connective tissue growth factor, CTGF）或内皮素-1（endothelin-1, ET-1）轴以及肿瘤坏死因子-α（tumor necrosis factor-α, TNF-α）来改善肺纤维化。

目前有几项正在进行的临床试验和临床前研究探讨不同分子在IPF发病机制中的作用，这些甚至在LC方面也有很好的应用前景；例如抗IL-13抗体（QAX576和Lebrikizumab），抗CCL2抗体（卡鲁单抗和CNTO-888）、抗TGF-β1抗体（FResolimumab和GC1008）、抗整合素αvβ6抗体（BG0011和STX-100）以及整合素αvβ6拮抗剂（GSK3008348）等^[58]^。

### 3.4 放射治疗

IPF-LC患者预后较差，患放射性肺炎的风险明显增加。如果这些患者要接受胸部放射治疗，则应仔细选择患者并严格选择适量的肺剂量-体积限制^[65]^。总之，IPF-LC在治疗上需紧密结合患者IPF的严重程度、IPF急性加重的风险、LC的病理分型及临床分期，选择个体化的治疗方案。

## 4 未来展望

IPF患者发生LC明显降低患者生存率及生存质量，给患者带来了更多痛苦。尽管Pirfenidone和Nintedanib两种已获批的抗纤维化药物可延长IPF患者的生存时间，降低LC的发生率，但目前仍无有效的治疗方法。IPF-LC的治疗目前缺乏有效的治疗方案，深入了解其细胞分子机制，针对其共同致病机制开发特异性药物或联合用药可能会是未来有效治疗此疾病的策略。本文希望通过对最新的IPF-LC的发病机制及治疗进行综述，能提高对IPF-LC发病机制的认识。
